# Genome-Wide Analysis of *Dof* Genes and Their Response to Abiotic Stress in Rose (*Rosa chinensis*)

**DOI:** 10.3389/fgene.2021.538733

**Published:** 2021-03-04

**Authors:** Hong Nan, Richard A. Ludlow, Min Lu, Huaming An

**Affiliations:** ^1^College of Agriculture, Guizhou University, Guiyang, China; ^2^School of Biosciences, Cardiff University, Cardiff, United Kingdom

**Keywords:** *Dof* transcription factor, rose (*Rosa chinensis*), phylogenetic analysis, synteny analysis, expression divergence, salt and drought stress

## Abstract

Dof (DNA binding with one finger) proteins play important roles in plant development and defense regulatory networks. In the present study, we report a genome-wide analysis of rose *Dof* genes (*RchDof*), including phylogenetic inferences, gene structures, chromosomal locations, gene duplications, and expression diversity. A total of 24 full-length *RchDof* genes were identified in *Rosa chinensis*, which were assigned to nine distinct subgroups. These *RchDof* genes were unevenly distributed on rose chromosomes. The genome-scale analysis of synteny indicated that segmental duplication events may have played a major role in the evolution of the *RchDof* gene family. Analysis of *cis*-acting elements revealed putative functions of *Dofs* in rose during development as well as under numerous biotic and abiotic stress conditions. Moreover, the expression profiles derived from qRT-PCR experiments demonstrated distinct expression patterns in various tissues, and gene expression divergence existed among the duplicated *RchDof* genes, suggesting a fundamentally functional divergence of the duplicated *Dof* paralogs in rose. The gene expression analysis of *RchDof*s under drought and salt stress conditions was also performed. The present study offered novel insights into the evolution of *RchDof*s and can aid in the further functional characterization of its candidate genes.

## Introduction

Plants have developed diverse molecular mechanisms to survive against various types of biotic and abiotic stress conditions. Numerous transcription factors have been identified in plants, which confer tolerance to a broad range of stress conditions. They are important regulators for adjusting gene expression by binding to specific DNA sequences at their promoter region (Wray et al., 2003). The Dof (DNA binding with one finger) gene family is one of the plant-specific transcription factors that is widespread in higher plants. Since the first *Dof* gene (*Zmdof1*) was isolated from maize (Yanagisawa and Izui, 1993), numerous *Dof* genes have been studied in other plants (Jin et al., 2014; Yang et al., 2018; Zhang et al., 2018; Hong et al., 2019).

The *Dof* gene family contains a highly conserved Dof domain at the N-terminus of approximately 52 residues in length. The Dof domain has a C2–C2 finger structure (CX_2_CX_21_CX_2_C) that can specifically bind to a core sequence (AT/AAAAG) in plant gene promoters and regulate downstream genes (Yanagisawa, 2002; Wang et al., 2019). However, some Dof proteins, such as *AOBP* in pumpkin, can bind to AGTA motif but have lost the capability to interact with the AT/AAAAG motif (Kisu et al., 1998). In addition to the Dof domain, Dof proteins harbor a bipartite nuclear localization signal that partly overlaps with the conserved Dof domain (Krebs et al., 2010) and a variable C-terminal transcriptional regulation domain (Yanagisawa, 2001). Moreover, the Dof domain and some specific amino acids in the C-terminal region also bind to specific DNA sequences to regulate various physiological activities (Yanagisawa and Schmidt, 1999).

DNA binding with one finger members have been reported to participate in the regulation of gene expression in diverse physiological processes. For example, the Dof transcription factor *MdDof24* identified in apple was reported to be associated with flower development and the regulation of metabolic pathways (Yang et al., 2018). *PbDof9.2* in pear (*Pyrus bretschneideri*) was reported to regulate flowering time. Overexpression of *PbDof9.2* in *Arabidopsis* could delay flowering time *via* interactions with the promoters of *PbTFL1a* and *PbTFL1b* (Liu et al., 2020). The peach Dof transcription factor *FaDof2* positively regulates eugenol biosynthesis by interacting with *FaEOBII* (Molina-Hidalgo et al., 2017). In addition, Dof transcription factors are also involved in abiotic and biotic stress responses, including heat, salt, drought, and pathogen attack. A total of 60 *Dof* genes were recently identified in the apple genome, and the expression levels of most *MdDof* members were upregulated by heat and salt stress conditions, revealing the important function of *Dof* genes in abiotic stress tolerance (Zhang et al., 2018). Overexpression of tomato *SlCDF1* (*SlDof25*) and *SlCDF3* (*SlDof26*) genes in *Arabidopsis* can increase tolerance to salt and drought stress (Corrales et al., 2014). The transient expression of *BBF1*-related *Dof* genes in tobacco enhanced the expression profiles of the mosaic viral resistance gene *N* and defense-related genes (Sasaki et al., 2015).

The Chinese rose (*Rosa chinensis* Jacq.) is an economically important flower crop of the Rosaceae family, which is widely cultivated in China (Feng et al., 2015). Although the cultivation of *R. chinensis* is increasing, it suffers from various biotic and abiotic conditions of stress. Drought and salt stress, which limit the growth and productivity of *R. chinensis*, had become the most harmful factors in the irrigated areas of China (Tian et al., 2018, 2019). Therefore, a better understanding of the molecular basis of drought and salt tolerance is required to breed new varieties with desirable traits. Despite the important role of *Dof* genes in plant drought and salt stress resistance, their exact functions have not yet been well studied in rose. The recently sequenced rose genome provides a framework for the identification and functional characterization of gene families (Raymond et al., 2018). Here we comprehensively characterized the number, structure, chromosomal locations, and phylogenetic associations of the *Dof* gene family throughout the rose genome. We also examined the expression differences of *Dof* genes in different tissues and in response to drought and salt stress conditions. The present study will form the foundation for further functional analysis of the *Dof* genes in rose.

## Materials and Methods

### Identification of Putative *Dof* Genes

The *Dof* genes of *A. thaliana* were obtained from tair^1^. The rose (*R. chinensis* “Old Blush”) genome sequences were downloaded from a rose website^2^. To comprehensively identify the *Dof* genes, the HMM file (PF02701) of the Dof domain was obtained from the Pfam database^3^ and used to perform the HMMER search (version 3.3^4^) with an *E*-value < 1e^–5^. The resulting *Dof* sequences were then adopted for TBLASTN as described before with default parameters (Altschul et al., 1997; Zou et al., 2019). Finally, following the removal of incorrect and redundant predicted sequences, the sequences of all candidate *Dofs* were further confirmed using ScanProsite^5^ and InterProScan^6^. The molecular weight (MW) and isoelectric point (pI) of the Dof proteins were evaluated using the ExPASy-ProtParam online software^7^. The subcellular localization of the Dof proteins was predicted using Plant-mPLoc^8^ (Chou and Shen, 2010). As a control, the apple and pear genome sequences were downloaded from GDR^9^ and Pear Genome Project^10^, respectively. The *Dof* transcription factors of these two Rosaceae species were also identified using the same method as described above.

### Phylogenetic Analysis of *RchDofs*

To investigate the phylogenetic associations among *Dofs*, a multiple sequence alignment including RchDof protein sequences and those from *Arabidopsis*, apple, and pear was performed using MUSCLE with default parameters in MEGA7 (Kumar et al., 2016). Subsequently, a maximum likelihood (ML) tree based on the above-mentioned alignment was constructed. The reliability of the obtained phylogenetic tree was tested using a bootstrap value of 1,000 iterations. To further determine the best-fit substitution model for the phylogeny tree, the ProtTest program (version 3.4) was used. Based on the multiple alignments of the Dof proteins and the classification of Lijavetzky, the *RchDof* genes were assigned to nine subgroups (Lijavetzky et al., 2003).

### Gene Structure Analysis and Identification of Conserved Motifs

To understand the structures of the *RchDof* genes, the GSDS (version 2.0^11^) online software was used to characterize the exon–intron structures. The motifs of each deduced RchDof protein were analyzed by MEME (version 4.12.0^12^) (Bailey et al., 2009), with the maximum number set to 30. The 1,500-bp upstream sequences of the *RchDof* genes were extracted with an in-house Perl script to predict *cis*-elements using the PlantCARE^13^ (Lescot et al., 2002).

### Determination of Chromosomal Distribution, Gene Duplication, and Synteny

The chromosomal location information of *RchDof*s was obtained from the GFF3 file. The synteny, segmental duplication, and tandem duplication were analyzed using a previously reported method (Nan and Gao, 2019) and visualized (including gene positions) by Circos (version 0.69) (Krzywinski et al., 2009). To estimate the duplication events of *RchDof* genes, Ka and Ks values were measured using the maximum likelihood method implemented in codeml program (Yang, 2007). The Ks values were subsequently used to approximately date the duplication event according to *T* = Ks/2λ, assuming clock-like rates (λ) of synonymous substitution of 1.5 × 10^–8^ (Zhang et al., 2018).

### Ortholog *Dof* Gene Identification

The orthologs of the candidate *RchDof*s in *Arabidopsis* were identified using Ensembl Plants (release 46,^14^). In addition, the orthologs in the tomato plant for each *RchDof* gene were analyzed as described previously (Nan and Gao, 2019), and the well-categorized tomato *Dof* sequences were obtained from Corrales (Corrales et al., 2014).

### Plant Material and Treatments

In this study, 3-year-old *R. chinensis* “Old Blush” plants were used as experimental materials, which were grown in the greenhouse at 22/18°C day/night temperature and 16/8 h day/night photoperiod. Two tissues, including matured leaves and fully blooming flowers, were collected for tissue-specific gene expression analysis. The drought stress treatment was performed with 20% PEG600, and matured leaves were collected at 0, 2, 4, 8, and 24 h following treatments (Li et al., 2016, 2017). Rose plants grown without drought stress were used as an unstressed control. A salinity stress treatment was carried out by irrigating the plants with 200 mM NaCl, followed by sampling matured leaves at 0, 2, 4, 8, and 24 h following treatments (Li et al., 2016, 2017). The plants irrigated with sterile water were used as a control. All samples were immediately frozen in liquid nitrogen and stored at −80°C until use. Three biological replicates were performed for each treatment.

### RNA Isolation and Quantitative Real-Time Polymerase Chain Reaction

Total RNA was extracted with Trizol reagent (Invitrogen) and treated with RNase-free DNase I (Transgen). Subsequently, 1 μg of total RNA was reverse-transcribed to cDNA using an All-in-One First-Strand cDNA Synthesis Kit (Transgen) according to the manufacturer’s instructions. Gene-specific primers of the *Dof* genes were designed using the Primer Premier 5.0 software and are presented in Supplementary Table 1. The *GAPDH* gene was selected as a reference gene according to a previous study (Tian et al., 2018), and three technical replicates were conducted for each sample. Real-time PCR was performed with a CFX96 real-time PCR detection system (Bio-Rad). Each reaction was carried out in a final volume of 10 μl. The reaction mixture contained the following reagents: 1.0 μl cDNA, 0.4 μl of each primer pair, 5 μl SYBR, and 3.6 μl ddH_2_O. The RT-PCR cycle was set as follows: 95°C for 30 s, followed by 40 cycles of 95°C for 5 s, and a final extension at 60°C for 30 s. A melting curve analysis for assessing specific amplification was performed by heating the products from 65°C to 95°C with 0.5°C increments. The 2^–Δ^ ^Δ^ ^CT^ method (Livak and Schmittgen, 2001) was used to analyze real-time PCR data, and the time point 0 h was used as an untreated control (expression = 1.0) to estimate the fold change in the expression levels of the relevant genes.

### Statistical Analyses

The experiment was performed in three biological replicates. All data were expressed as mean ± standard deviations (SD) following normalization. Statistical analysis was performed using SPSS software (version 18.0). Data were analyzed using Fisher’s least significant difference analysis, and significant differences were reported at *P* < 0.05 level.

## Results

### The *Dof* Gene Family in the Rose Genome

**FIGURE 1:**
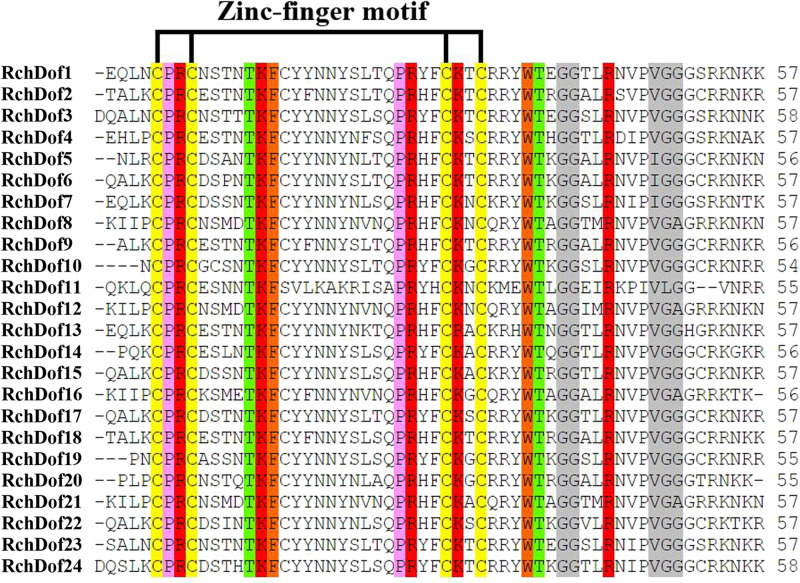
Dof domain sequence alignment of rose Dof proteins. Only the conserved Dof domain residue is currently shown. The four cysteine residues putatively responsible of the zinc-finger structure are indicated. Identical and similar amino acids are highlighted.

Using the consensus sequences of the Dof domains, we screened the rose genome assembly. We identified 24 non-redundant *RchDof* genes and named them as *RchDof1* to *RchDof24* based on the order of the gene IDs (Supplementary Table 2). The open reading frame lengths of the *RchDof* genes ranged from 173 to 531 amino acids, with the p*I* of the resultant proteins predicted to range from 4.82 to 9.48 and the MW from 19.52 to 56.83 kDa (Supplementary Table 2). The predicted grand average of hydropathicity values of the RchDof proteins varied from −0.888 (RchDof14) to −0.397 (RchDof4), suggesting that they were hydrophilic. Furthermore, the predicted subcellular localizations indicated that all RchDof proteins were located in the nucleus. In addition, multiple sequence alignment of RchDofs revealed a highly conserved Cys2/Cys2 Zn^2+^ DNA binding domain (Figure 1), which was designated as the Dof domain. The distribution of amino acid residues at the corresponding positions of the rose Dof domain revealed that it was very similar to that of *Arabidopsis* (Lijavetzky et al., 2003), apple (Hong et al., 2019), tomato (Cai et al., 2013), pear (Liu et al., 2020), cassava (Zou et al., 2019), and pepper (Wu et al., 2016), indicating that the Dof domain was highly conserved among different higher plants. The detailed information of these *RchDof* genes regarding the type of genes and the Dof domains is shown in Supplementary Table 2 and Figure 1.

### Phylogenetic Analysis of *RchDof* Genes

**FIGURE 2:**
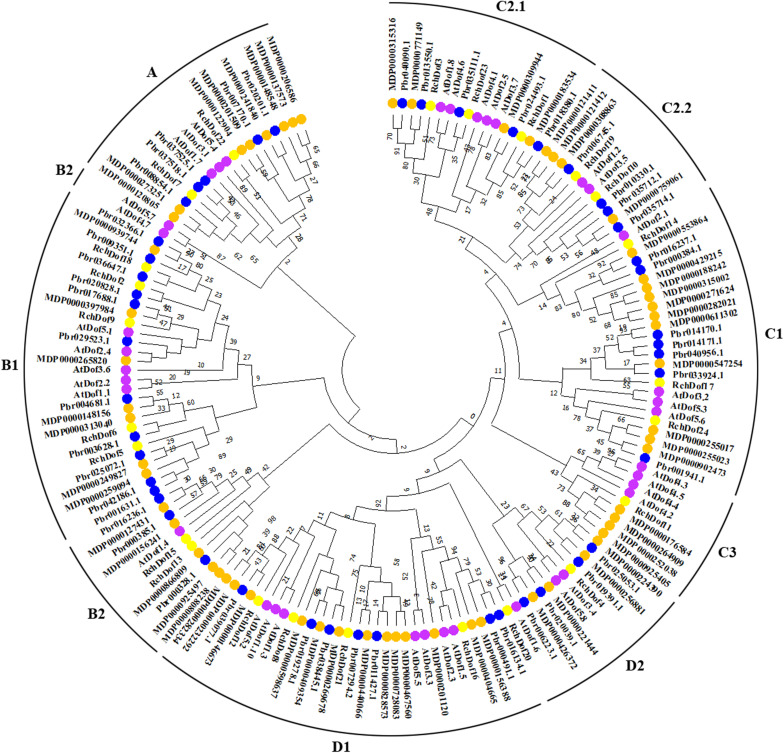
Phylogenetic tree based on Dof proteins from rose, *Arabidopsis*, apple, and pear. The phylogenetic tree was constructed using the maximum likelihood method. The reliability of the predicted tree was tested using bootstrapping with 1,000 replicates.

To further examine the evolutionary relationships in the *Dof* genes, an un-rooted ML phylogenetic tree was constructed using *R. chinensis* and other species (*Arabidopsis*, apple, and pear). Using the ProtTest program, we found that JTT + G was the best substitution model. As shown in Figure 2, the Dof proteins in the four species were classified into nine groups, namely, A, B1, B2, C1, C2.1, C2.2, C3, D1, and D2, respectively. The 24 *RchDof* genes were unevenly distributed in the nine subgroups. Class B1 was the largest subfamily, which contained five *RchDof* factors. Class C3 was the smallest class, containing only one member. Furthermore, classes A, B2, C2.2, and D2 were present at the same proportion of 8.33% (Figure 2). The phylogenetic tree showed that all the nine classes were monophyletic, except for B2. Group B2 could be divided into two clades, one of which was the largest and clustered with class B1, and the other was clustered with class A. These results were consistent with the past results of physic nut and castor bean (Zou and Zhang, 2019). In addition, the phylogenetic analysis also indicated that most *Dof* genes of rose were clustered with the *Dof* members of apple and pear, suggesting a close relationship among the three Rosaceae species. When the number of *Dof* genes was compared among these three species, we observed that the number in apple and pear was greatly increased by almost 2.5 and 1.9 times than that of rose. The results indicated that a large-scale expansion of *Dof* members in apple and pear seemingly occurred after the divergence of the three Rosaceae species, evidenced by the *Dof* members of apple and pear exclusively clustered together in most groups, respectively.

### Protein Structure of the *RchDof* Gene Family

**FIGURE 3:**
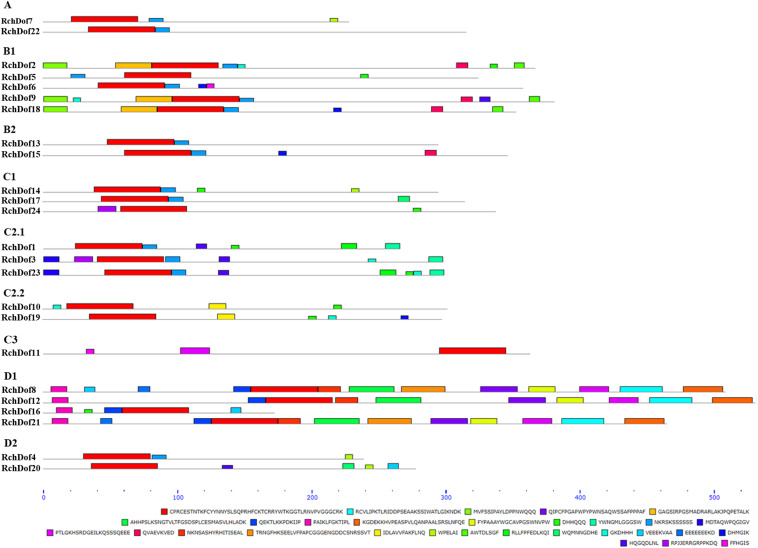
Conserved motif compositions of the *RchDof* gene family. The conserved motifs were detected using MEME software and represented by colored boxes. The length of RchDof proteins can be estimated using the scale at the bottom, and the conserved motifs are shown in Table 1.

To reveal the structural variation of the *RchDof* genes in rose, we predicted putative motifs using the program MEME (Bailey et al., 2009) and identified a total of 30 distinct motifs. The schematic distribution of these motifs among different gene groups is described (Figure 3), representing their relative locations within the proteins. The multi-level consensus sequences were produced among these motifs (Table 1). Among the 30 identified motifs, motif 1 was considered the Dof domain and was uniformly observed across all the RchDof proteins. In contrast to motif 1, majority of subgroups of RchDofs exhibited several special motifs at their C-terminal regions, and little was known about these structures. Motifs 16 and 21 were widely present in most members of group B1, motif 12 was limited to group C2.1, motif 19 was present in all members of group C2.2, motif 9 was limited to group D1, and motif 20 was present in all members of group D2 (Figure 3). Moreover, as expected, the majority of closely associated members in the phylogenetic tree possessed common motif compositions, suggesting a functional similarity among the Dof proteins within the same subfamily (Figure 3). Almost all members in group C2.1 possessed motifs 12, 13, and 28, group B1 usually contained motifs 3, 5, 13, 16, and 21, and group D1 harbored motifs 8, 7, 17, 6, 4, 10, 15, 2, and 9 in order. The motif distribution differences among the different groups or subgroups indicated the functional divergence of the *Dof* gene family. The protein structure of the *Dof* genes corroborated with the ML phylogenetic tree.

**TABLE 1:** Conserved motifs of RchDof proteins in the *Rosa* genome.

Motif ID	Conservative motifs	E-value	Width	Sites	Description
Motif 1	CPRCESTNTKFCYYNNYSLSQPRHFCKTCRRYWTKGGTLRNVPVGGGCRK	6.6e − 1,009	50	24	WDP^*a*^
Motif 2	RCVLIPKTLRIDDPSEAAKSSIWATLGIKNDK	3.4e − 026	32	3	
Motif 3	MVFSSIPAYLDPPNWQQQ	1.4e − 013	18	3	
Motif 4	QIPCFPGAPWPYPWNSAQWSSAFPPPAF	4.9e − 012	28	3	
Motif 5	GAGSIRPGSMADRARLAKJPQPETALK	1.3e − 011	27	3	
Motif 6	AHHPSLKSNGTVLTFGSDSPLCESMASVLHLADK	2.3e − 011	34	3	
Motif 7	QEKTLKKPDKIJP	1.1e − 009	13	4	
Motif 8	PAIKLFGKTIPL	8.5e − 009	12	4	
Motif 9	KGDEKKHVPEASPVLQANPAALSRSLNFQE	5.8e − 010	30	3	
Motif 10	FYPAAAYWGCAVPGSWNVPW	2.4e − 006	20	3	
Motif 11	DHHQQQ	1.7e − 005	6	9	
Motif 12	YWNGMLGGGSW	1.1e − 002	11	3	
Motif 13	NKRSKSSSSSS	6.0e − 002	11	15	
Motif 14	MDTAQWPQGIGV	1.6e − 001	12	2	
Motif 15	PTLGKHSRDGEILKQSSSQEEE	1.7e + 000	22	4	
Motif 16	QVAEVKVED	2.1e + 000	9	4	
Motif 17	NKNSASHYRHITISEAL	2.3e + 000	17	3	
Motif 18	TRNGFHKSEELVFPAPCGGGENGDDCSNRSSVT	5.3e + 000	33	2	
Motif 19	IDLAVVFAKFLNQ	1.2e + 001	13	2	
Motif 20	WPELAI	1.2e + 001	6	4	
Motif 21	AWTDLSGF	1.4e + 001	8	3	
Motif 22	RLLFPFEDLKQI	3.4e + 001	12	2	
Motif 23	WQMNNGDHE	4.4e + 001	9	2	
Motif 24	GKDHHH	2.3e + 001	6	6	
Motif 25	VEEEKVAA	9.8e + 001	8	3	
Motif 26	EEEEEEEKD	1.7e + 002	9	2	
Motif 27	DHMGIK	1.4e + 002	6	4	
Motif 28	HQGQDLNL	2.6e + 002	8	5	
Motif 29	RPJJERRGRPPKDQ	4.7e + 002	14	2	
Motif 30	FFHGIS	5.4e + 002	6	2	

*^*a*^WDP indicates a part of Dof domain.*

### Exon–Intron Organization of the *RchDof* Genes

**FIGURE 4:**
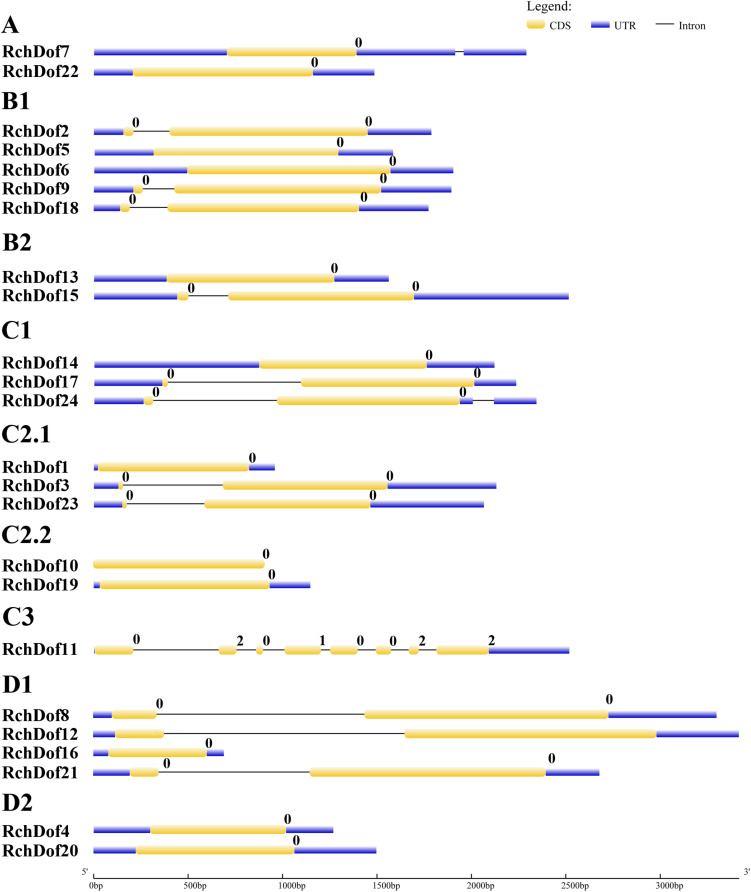
Exon–intron structures of *RchDof* genes. The exons and introns are indicated by yellow rectangles and black lines, respectively. The length of RchDof proteins can be estimated using the scale at the bottom. The intron phases are indicated as numbers 0, 1, and 2.

To further examine the evolution of the *RchDof* genes, we investigated their exon–intron structures. As illustrated in Figure 4, the exon numbers of *RchDof* genes ranged from one to eight. Nearly all the *RchDof* genes exhibited no intron or a single intron, except for *RchDof11*. Similar exon–intron structures were also observed in the *Dof* genes of *Arabidopsis*, rice, castor bean, and cassava (Lijavetzky et al., 2003; Jin et al., 2014; Zou et al., 2019). Moreover, the data showed that the *RchDof* genes in the same group exhibited similar exon–intron compositions. For example, the majority of C2.1 and D1 contained one intron, whereas groups C2.2 and D2 had no intron. Interestingly, we found that there were no significant differences among *RchDof* genes in the intron phase, except for *RchDof11*, which contained phase 0, 2, 0, 1, 0, 0, 2, and 2 introns.

### Stress-Related *Cis*-Elements in Promoters of *RchDof* Genes

In order to investigate the evolution and functional divergence of the *RchDof* genes, the upstream 1.5-kb promoter regions of all the *RchDof* members were extracted and analyzed using the PlantCARE online software. Various *cis*-acting regulatory elements were analyzed, including 10 elements related to plant development and 11 motifs associated with stress response (Supplementary Table 3). As shown in Supplementary Table 3, all members contained more than one *cis*-element, and the majority of the *RchDof* genes possessed box4, G-box, CGTCA motif, and ABRE. It is interesting to note that two *cis*-regulation elements, including the ACE and GARE motif, were only contained in *RchDof8* and *RchDof4*, respectively. A total of eight *RchDof*s possessed the W-box (TTGACC), which regulates gene expression by binding to the WRKY transcription factors, suggesting that these genes may be cross-regulated by other proteins. MBS is a MYB-binding site involved in drought response and was identified in eight genes, indicating that these *RchDofs* participated in drought stress response.

### Chromosomal Location, Gene Duplication, and Genomic Synteny of *RchDof* Genes

**FIGURE 5:**
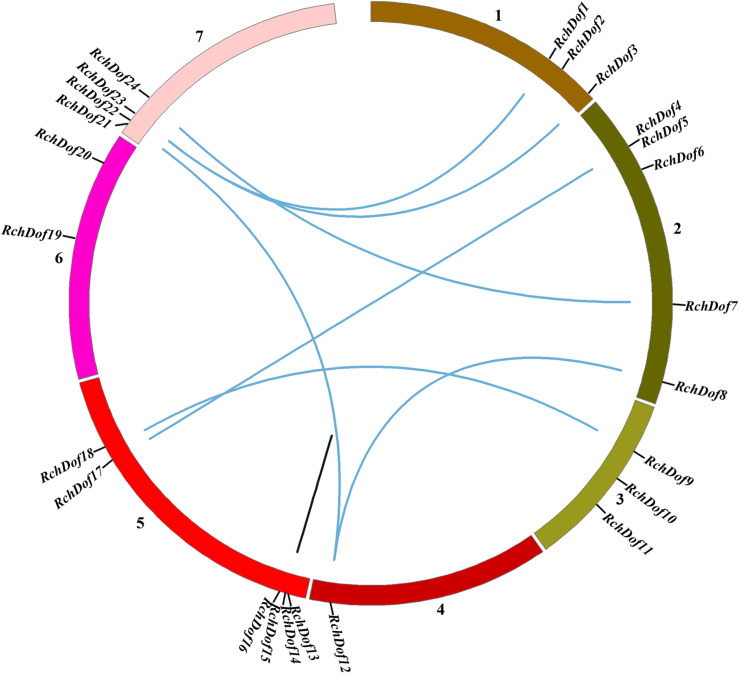
Chromosome distribution and synteny analysis of rose *Dof* genes. Chromosomes 1–7 are shown with different colors. The approximate location of each *RchDof* gene is marked with a short black line. Blue lines indicate segmentally duplicated *RchDof* genes, and black lines in the circle indicate tandemly duplicated genes.

To examine the genomic distribution of the *RchDof* genes, their chromosomal locations were searched against the rose genome database. The results indicated that 24 *RchDof*s were unevenly distributed on the seven pseudo-chromosomes. As shown in Figure 5, chromosome 5 exhibited the largest number of the *RchDof* genes, followed by chromosome 2, which had five, and chromosome 4 that harbored only one.

It has been reported that gene duplication events are a primary source of genetic novelty and the main effect in gene family expansion (Moore and Purugganan, 2005). Duplications are classified into three types: segmental, tandem, and dispersed (Li et al., 2012). In order to trace the origins of the *RchDof* genes in rose, we performed syntenic analysis with MCScanX. The collinear relationships of the duplicated pairs in the *RchDof* genes are shown in Table 2. Out of the 24 *RchDof* genes, seven pairs (12 *RchDof* genes) might have resulted from segmental duplication events, while the remaining one cluster (*RchDof14* and *RchDof15*) probably originated from tandem duplication events. The results indicate that segmental duplication has played a predominant role in the evolution of the *RchDof* genes. In other plants, the majority of *Dof* genes were also found to derive from segmental duplication events, such as in apple (Zhang et al., 2018), cassava (Zou et al., 2019), tomato (Cai et al., 2013), and cotton (Li et al., 2018). In addition, the majority of the segmentally duplicated genes belonged to the same *RchDof* subgroup, with the exception of two clusters (*RchDof5*/*RchDof17* and *RchDof7*/*RchDof24*) (Table 2). This phenomenon may result from the gain or loss of certain motif structures. As shown in Figure 5, chromosomes 2 and 7 contained the most segmentally duplicated *RchDof* genes, while chromosome 6 harbored none. The tandem-duplicated gene pair was located on chromosome 5. In addition, the two tandem-duplicated genes in one pair belonged to different classes (Table 2). These results indicate that the tandem-duplicated pairs may have undergone functional divergence.

**TABLE 2:** Ka/Ks calculation and divergence times of the duplicated *RchDof* gene pairs in syntenic blocks.

Duplicated gene pairs		Group	Ka	Ks	Ka/Ks	Duplicated type	Date (Mya)
*RchDof1*	*RchDof23*	C2.1	0.39	1.27	0.30	WGD	42.40
*RchDof3*	*RchDof23*	C2.1	0.42	1.25	0.34	WGD	41.58
*RchDof8*	*RchDof12*	D1	0.33	1.40	0.24	WGD	46.76
*RchDof5*	*RchDof17*	B1/C1	1.09	3.17	0.34	WGD	105.76
*RchDof7*	*RchDof24*	A/C1	0.69	3.19	0.22	WGD	106.39
*RchDof9*	*RchDof18*	B1	0.51	2.11	0.24	WGD	70.46
*RchDof12*	*RchDof21*	D1	0.37	1.10	0.33	WGD	36.79
*RchDof14*	*RchDof15*	C1/B2	0.97	3.55	0.27	Tandem	118.37

*Ka, non-synonymous substitution rate; Ks, synonymous substitution rate; Mya, millions of years ago; WGD, segmental duplication.*

As the ratio Ka/Ks is a good indicator of the selective pressure occurring at the protein level, we used the PAML software to estimate the Ks (synonymous) and Ka (non-synonymous) values as well as the Ka/Ks ratio. These duplication events were approximately dated using the formula: *T* = Ks/2r (Table 2). The duplication-derived *RchDof* genes spanned from 36.79 to 118.37 Mya (millions of years ago). It is often assumed that the values Ka/Ks < 1, Ka/Ks = 1, and Ka/Ks > 1 indicate negative selection, neutral evolution, and positive selection, respectively (Li, 1993; Yang and Bielawski, 2000). All duplicated *RchDof*s from the seven segmentally duplicated gene pairs had Ka/Ks < 1, ranging from 0.22 to 0.34, while the Ka/Ks of the tandem-duplicated gene pair was 0.27. The results suggest that all of the duplicated gene pairs are under strong purifying selection, which corroborates with observations in other plants, such as apple and tomato (Cai et al., 2013; Zhang et al., 2018).

### Profiling of *RchDof* Gene Expression

**FIGURE 6:**
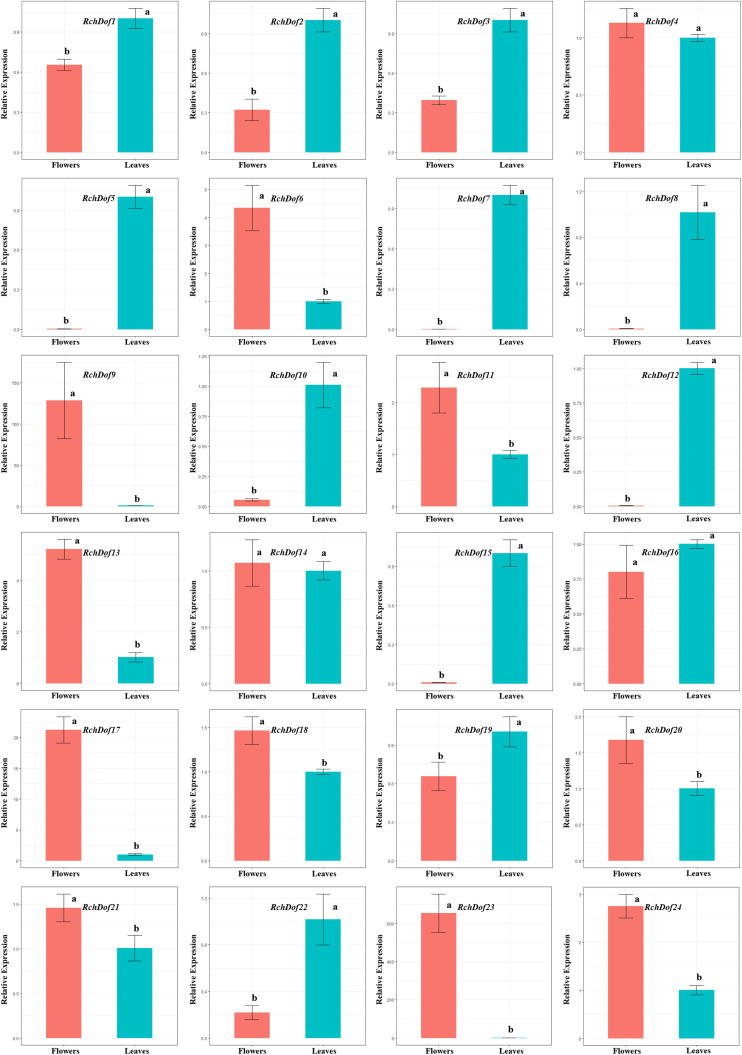
Expression patterns of *RchDof* genes in tissues of rose. The x-axis shows different tissues, while the y-axis represents the relative expression levels of *RchDof* genes compared with *GAPDH* gene using 2^− ΔΔCT^ method. Error bars indicate the standard deviations (mean ± SD) of three independent replicates. The histogram bars labeled with different letters (a and b) above them are significantly different (least significant difference test, *P* < 0.05).

The expression patterns of the 24 *RchDof* genes in leaves and flowers were examined using real-time reverse transcription-PCR (qRT-PCR) experiments. The primer pairs for all genes are listed in Supplementary Table 1. The results indicated that the *RchDof* genes were differentially expressed in these two tissues (Figure 6). Some *RchDof* genes, such as *RchDof5* (group B1), *RchDof7* (group A), *RchDof8* (group D1), *RchDof12* (group D1), and *RchDof15* (group B2), were highly expressed at maximum levels in leaves. The remaining *RchDof* genes, such as *RchDof9* (group B1), *RchDof13* (group B2), and *RchDof17* (group C1), exhibited a low expression in leaves, whereas they were highly expressed in flowers. Interestingly, the results suggested that *RchDof* genes that were classified as of the same subgroup could exhibit distinctive expression patterns among tissues, such as *RchDof13* (group B2) and *RchDof15* (group B2). In addition, gene expression divergence was also investigated by comparing the levels of the duplicated *RchDof* genes. The results indicated that the expression divergence was also present among duplicated *RchDof* paralogs. For example, *RchDof1* and *RchDof23* were segmentally duplicated gene pairs. *RchDof1* expression was upregulated in the leaves and downregulated in the flowers, while the expression of its paralog *RchDof23* was downregulated in the leaves and upregulated in the flowers. Previous studies reported that tissue-specific expression divergence is one of the most important indicators of functional differentiation between genes (Makova and Li, 2003; Li et al., 2005). Therefore, the same subgroup and the expanded *Dof* genes may result in novel biological function during plant evolution, which is beneficial to regulate various physiological processes by removing their redundancy.

### Expression Profiling of *RchDof* Gene Under Drought and Salt Stress

The functions of the rose *Dof* genes in response to abiotic stress are largely unknown. In order to clarify the potential functions of the *RchDof* genes in response to abiotic stress conditions, we analyzed their expression patterns under specific stress conditions, including drought and salinity treatment. The analysis was performed using qRT-PCR in leaves.

**FIGURE 7:**
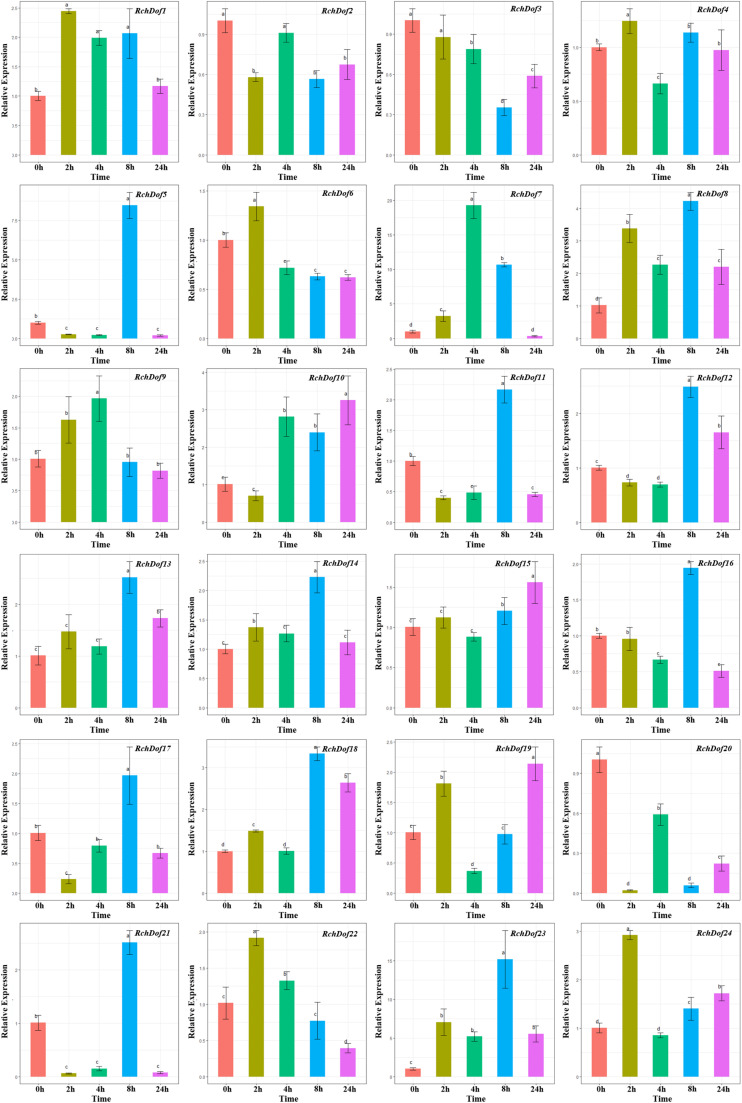
Expression patterns of *RchDof* genes under drought stress. Samples were collected at 0, 2, 4, 8, and 24 h after treatment. Error bars indicate the standard deviations (mean ± SD) of three independent replicates. The histogram bars labeled with different letters (a–d) above them are significantly different (least significant difference test, *P* < 0.05).

As shown in Figure 7, *RchDof* genes were sensitive to drought stress, the majority of which exhibited different expression patterns. Some genes were evidently up-regulated after 8 h and followed by a decrease, such as *RchDof5*, *RchDof11*, *RchDof12*, and *RchDof21*. Some were gradually induced, peaked at 2 h, and then decreased in 4, 8, and 24 h (*RchDof6* and *RchDof22*). The greatest increase in expression (nearly 19-fold) occurred for the *RchDof7* gene at 4 h. The results indicated that the expression levels of *RchDofs* were responsive to drought stress. The expression divergence was also examined by comparing the expression levels of the duplicated *RchDof* genes. For example, the pair of duplicated genes *RchDof9* and *RchDof18* was a case of segmental duplication. *RchDof9* indicated the higher expression following 4 h, whereas *RchDof18* reached the higher level in the leaves following 8 h.

**FIGURE 8:**
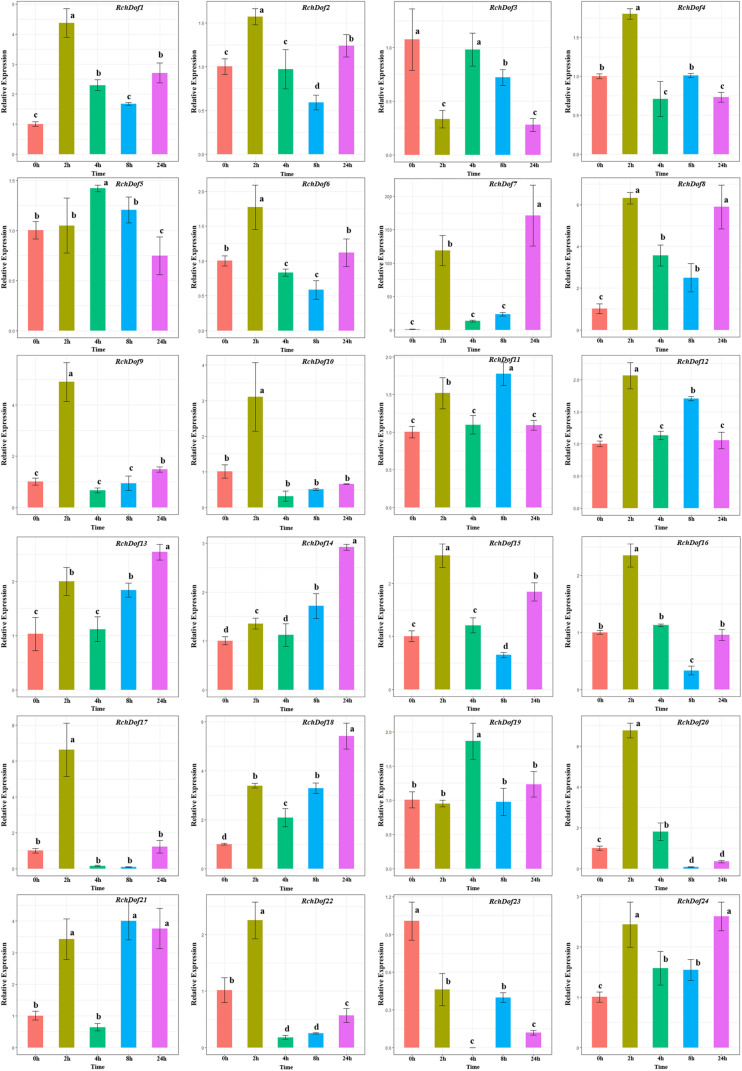
Expression patterns of *RchDof* genes under salt stress. Samples were collected at 0, 2, 4, 8, and 24 h after treatment. Error bars indicate the standard deviations (mean ± SD) of three independent replicates. The histogram bars labeled with different letters (a–d) above them are significantly different (least significant difference test, *P* < 0.05).

To investigate the expression pattern of the *RchDof* genes under salt stress conditions, the plants were irrigated with 200 mM NaCl solution. Salt treatment resulted in a wide variety of *RchDof* gene expression profiles. As described in Figure 8, the gene expression levels of *RchDof9*, *RchDof10*, *RchDof17*, and *RchDof20* were rapidly increased by 4.8, 3, 6.6, and 6.7 times following 2 h, respectively. After that time period, they decreased. The expression levels of *RchDof23* sharply decreased at 2 h and reached the lowest levels after 4 h. In addition, the greatest increase in expression levels was noted for *RchDof7* (nearly 163-fold).

## Discussion

*Dof* genes play essential roles in various plant physiological processes as well as diverse abiotic and biotic stress responses (Cai et al., 2013; Corrales et al., 2014; Wu et al., 2016). Despite roses (*R. chinensis* Jacq.) being the most commercially important plant in the Rosaceae family, *Dof* genes in rose have not been comprehensively characterized, and their exact functions remain unknown. In the current study, a search for *Dof* genes in the rose genome resulted in the identification of 24 members, which were named from *RchDof1* to *RchDof24* based on their gene IDs. In addition, an analysis of their structure, duplication events, and expression diversity was conducted with regard to drought and salt stress.

Compared with rose (24 *RchDof*s, genome size 560 Mb) (Yokoya, 2000), a comparable number of *Dof*s was identified in castor bean (24 *Dof*s, genome size 320 Mb) (Chan et al., 2010; Zou and Zhang, 2019) and grape (25 *Dof*s, genome size 487 Mb) (Jaillon et al., 2007; da Silva et al., 2016), although the number of *Dofs* was greater in *Arabidopsis* (36 *Dof*s, genome size 125 Mb) (Yanagisawa, 2002; Ma et al., 2015) and Chinese cabbage (76 *Dof*s, genome size 485 Mb) (Ma et al., 2015). This suggests that the number of *Dof* genes is not associated with the genome size. The gene structure, protein composition, exon–intron organization, and phylogenetic relationships support the conclusion that these 24 RchDof proteins can be divided into four major groups (A, B, C, and D) as previously described in other plant species (Liu and Ekramoddoullah, 2009; Xiao et al., 2017). Furthermore, in the present study, class B2 could be obviously divided into two clusters (Figure 2). Thereby, we updated the classification as follows: class 1 (A), 2 (B1), 3 (B2), 4 (B2), 5 (C1), 6 (C2.1), 7 (C2.2), 8 (C3), 9 (D1), 10 (D2), and 11 (D2).

Gene duplication has long been regarded as a key contributor to plant gene evolution (Li et al., 2005). Our results indicate that seven pairs of *RchDof* duplicated genes were derived from segmental duplication events, and one pair of *Dof* genes was generated by tandem duplications. A number of studies suggested that genes arising through segmental duplication events may more often be retained due to sub-functionalization without increasing the likelihood of gene rearrangement (Zhang, 2003). Segmental duplication may have played a more important role than tandem duplication in driving *Dof* gene family evolution, as suggested by previous findings in *A. thaliana*, rice, and apple (Cannon et al., 2004; Wang et al., 2014; Huang et al., 2015). The spatiotemporal expression changes are the main indicators of functional divergence in duplicated genes (Makova and Li, 2003; Hellsten et al., 2007). The 24 candidate *RchDof* genes in leaves and flowers displayed markedly different expression profiles, of which even genes in the same subgroup were divergently expressed. It is interesting to note that six pairs of duplicated genes (*RchDof1/RchDof23*, *RchDof3/RchDof23*, *RchDof5/RchDof17*, *RchDof7/RchDof24*, *RchDof9/RchDof18*, and *RchDof12/RchDof21*) arising from segmental duplication events exhibited exceptionally different expression patterns, indicating that the functional divergence may have provided genetic sources with novel biological functions during the evolution of the *RchDof* gene family. These results indicate that the expanded *RchDof* genes might result in novel biological complexity in order to remove function redundancy.

Drought and high salinity cause abiotic stress that influences plant growth and development. However, as described previously, roses are often grown under non-stressed conditions, and natural selection has unintentionally narrowed the genetic variability of abiotic stress tolerance. Therefore, the understanding of the mechanism by which roses respond and develop tolerance to drought and salt stress is the first step toward improving the adaptation of commercial rose cultivars to stressful environments. It was reported that *Dof* genes were involved in a wide variety of biological processes, including drought and salt stress responses (Corrales et al., 2014; Cai et al., 2016; Renau-Morata et al., 2017). Here we demonstrated that the expression levels of all the *RchDof* genes were upregulated/downregulated following drought and salinity treatments, suggesting that they may be involved in drought and salt stress responses. These results are supported by previous findings, as *CDF3* (AT3G47500), the orthologs gene of *RchDof21* (Supplementary Table 2), is well studied for its role in salinity stress (Renau-Morata et al., 2017). In addition, the orthologs gene of *RchDof12* and *RchDof21* in tomato (*SlCDF1*) (Supplementary Table 2) demonstrated increased drought and salt tolerance (Corrales et al., 2014). Therefore, our findings suggest that rose *Dof* genes have a role in response to drought and salt stress. The functional characterization of *RchDof12* and *RchDof21* will aid in the exploration of the mechanism of drought and salt tolerance in future studies. Moreover, in contrast to *RchDof12* and *RchDof21*, *RchDof7* showed the highest increase in expression following drought and salinity treatment. *Cis*-element analysis indicated that the *RchDof7* exhibited a MYB binding site, which is involved in the drought response. These results support our hypothesis that the *RchDof7* gene may play a key role in drought and salt responses. The results also provide a number of *RchDof* candidate genes for improving drought and salt tolerance in rose.

## Conclusion

In this study, a total of 24 *RchDof* genes were identified in the rose genome assembly. Phylogenetic analysis suggested that the *RchDof* transcription factors were conserved as demonstrated by the identification of highly conserved motifs and gene structures. Genomic synteny analysis suggested that segmental duplications may have played a major role in *RchDof* gene family evolution. The *cis-*element analysis suggested that the majority of the *RchDof* genes were involved in various processes as well as stress responses, which provides a basis for the functional characterization of *RchDof* genes. The data showed the tissue-specific expression of the *RchDofs* and differential expression in response to drought and salt stress conditions, suggesting the existence of a complicated molecular regulatory network response to drought and salt stress conditions in rose. This provides novel insights into the evolutionary and functional divergence of the *Dof* gene family, which can aid in functional genomic studies of candidate *Dof* genes in order to genetically improve commercially important rose cultivars.

## Data Availability Statement

The raw data supporting the conclusions of this article will be made available by the authors, without undue reservation.

## Author Contributions

HN and ML performed the data analysis and experiments. HN drafted the manuscript. ML and HA served as the principal investigator, facilitated the project, and revised the manuscript. RL revised the manuscript. All authors contributed to the article and approved the submitted version.

## Conflict of Interest

The authors declare that the research was conducted in the absence of any commercial or financial relationships that could be construed as a potential conflict of interest.

## Footnotes

1 https://www.arabidopsis.org/

2 https://lipm-browsers.toulouse.inra.fr/pub/RchiOBHm-V2/

3 http://pfam.xfam.org/

4 http://hmmer.janelia.org/

5 http://www.expasy.ch/tools/scanprosite/

6 http://www.ebi.ac.uk/Tools/InterProScan/

7 http://web.expasy.org/protparam/

8 http://www.csbio.sjtu.edu.cn/bioinf/plant-multi/

9 https://www.rosaceae.org/

10 http://peargenome.njau.edu.cn

11 http://gsds.gao-lab.org/

12 http://meme-suite.org/tools/meme

13 http://bioinformatics.psb.ugent.be/webtools/plantcare/html/

14 http://plants.ensembl.org/index.html

## References

[B1] Altschul S. F. Madden T. L. Schäffer A. A. Zhang J. Zhang Z. Miller W. (1997). Gapped BLAST and PSI-BLAST: a new generation of protein database search programs. *Nucleic Acids Res.* 25 3389–3402. 9254694 10.1093/nar/25.17.3389 PMC146917

[B2] Bailey T. L. Boden M. Buske F. A. Frith M. Grant C. E. Clementi L. (2009). MEME SUITE: tools for motif discovery and searching. *Nucleic Acids Res.* 37(Suppl._2), W202–W208. 10.1093/nar/gkp335 19458158 PMC2703892

[B3] Cai X. Zhang C. Shu W. Ye Z. Li H. Zhang Y. (2016). The transcription factor SlDof22 involved in ascorbate accumulation and salinity stress in tomato. *Biochem. Biophs. Res. Commun.* 474 736–741. 10.1016/j.bbrc.2016.04.148 27157141

[B4] Cai X. Zhang Y. Zhang C. Zhang T. Hu T. Ye J. (2013). Genome-wide analysis of plant-specific Dof transcription factor family in tomato. *J. Integr. Plant Biol.* 55 552–566. 10.1111/jipb.12043 23462305

[B5] Cannon S. B. Mitra A. Baumgarten A. Young N. D. May G. (2004). The roles of segmental and tandem gene duplication in the evolution of large gene families in *Arabidopsis thaliana*. *BMC Plant Biol.* 4:10. 10.1186/1471-2229-4-10 15171794 PMC446195

[B6] Chan A. P. Crabtree J. Zhao Q. Lorenzi H. Orvis J. Puiu D. (2010). Draft genome sequence of the oilseed species *Ricinus communis*. *Nat. Biotechnol.* 28 951–956. 10.1038/nbt.1674 20729833 PMC2945230

[B7] Chou K. C. Shen H. B. (2010). Plant-mPLoc: a top-down strategy to augment the power for predicting plant protein subcellular localization. *PLoS One* 5:e11335. 10.1371/journal.pone.0011335 20596258 PMC2893129

[B8] Corrales A. R. Nebauer S. G. Carrillo L. Fernández-Nohales P. Marqués J. Renau-Morata B. (2014). Characterization of tomato cycling Dof factors reveals conserved and new functions in the control of flowering time and abiotic stress responses. *J. Exp. Bot.* 65 995–1012. 10.1093/jxb/ert451 24399177

[B9] da Silva D. C. da Silveira Falavigna V. Fasoli M. Buffon V. Porto D. D. Pappas G. J. Jr. (2016). Transcriptome analyses of the *Dof*-like gene family in grapevine reveal its involvement in berry, flower and seed development. *Hortic. Res.* 3:16042. 10.1038/hortres.2016.42 27610237 PMC5005469

[B10] Feng L. Ding H. Wang J. Wang M. Xia W. Zang S. (2015). Molecular cloning and expression analysis of *RrNHX1* and *RrVHA-c* genes related to salt tolerance in wild *Rosa rugosa*. *Saudi J. Biol. Sci.* 22 417–423. 10.1016/j.sjbs.2015.01.008 26150747 PMC4487260

[B11] Hellsten U. Khokha M. K. Grammer T. C. Harland R. M. Richardson P. Rokhsar D. S. (2007). Accelerated gene evolution and subfunctionalization in the pseudotetraploid frog *Xenopus laevis*. *BMC Biol.* 5:31. 10.1186/1741-7007-5-31 17651506 PMC1949811

[B12] Hong K. Xian J. Jia Z. Hou X. Zhang L. (2019). Genome-wide identification of Dof transcription factors possibly associated with internal browning of postharvest pineapple fruits. *Sci. Hortic.* 251 80–87. 10.1016/j.scienta.2019.03.007

[B13] Huang X. Li K. Xu X. Yao Z. Jin C. Zhang S. (2015). Genome-wide analysis of WRKY transcription factors in white pear (*Pyrus bretschneideri*) reveals evolution and patterns under drought stress. *BMC Genom.* 16:1104. 10.1186/s12864-015-2233-6 26704366 PMC4691019

[B14] Jaillon O. Aury J. M. Noel B. Policriti A. Clepet C. Casagrande A. (2007). The grapevine genome sequence suggests ancestral hexaploidization in major angiosperm phyla. *Nature* 449 463–467. 10.1038/nature06148 17721507

[B15] Jin Z. Chandrasekaran U. Liu A. (2014). Genome-wide analysis of the Dof transcription factors in castor bean (*Ricinus communis* L.). *Genes Genom.* 36 527–537. 10.1007/s13258-014-0189-6

[B16] Kisu Y. Ono T. Shimofurutani N. Suzuki M. Esaka M. (1998). Characterization and expression of a new class of zinc finger protein that binds to silencer region of ascorbate oxidase gene. *Plant Cell Physiol.* 39 1054–1064. 10.1093/oxfordjournals.pcp.a029302 9871365

[B17] Krebs J. Mueller-Roeber B. Ruzicic S. (2010). A novel bipartite nuclear localization signal with an atypically long linker in DOF transcription factors. *J. Plant Physiol.* 167 583–586. 10.1016/j.jplph.2009.11.016 20116130

[B18] Krzywinski M. Schein J. Birol I. Connors J. Gascoyne R. Horsman D. (2009). Circos: an information aesthetic for comparative genomics. *Genome Res.* 19 1639–1645. 10.1101/gr.092759.109 19541911 PMC2752132

[B19] Kumar S. Stecher G. Tamura K. (2016). MEGA7: molecular evolutionary genetics analysis version 7.0 for bigger datasets. *Mol. Biol. Evol.* 33 1870–1874. 10.1093/molbev/msw054 27004904 PMC8210823

[B20] Lescot M. Déhais P. Thijs G. Marchal K. Moreau Y. Van de Peer Y. (2002). PlantCARE, a database of plant *cis*-acting regulatory elements and a portal to tools for in silico analysis of promoter sequences. *Nucleic Acids Res.* 30 325–327. 11752327 10.1093/nar/30.1.325 PMC99092

[B21] Li H. Dou L. Li W. Wang P. Zhao Q. Xi R. (2018). Genome-wide identification and expression analysis of the Dof transcription factor gene family in *Gossypium hirsutum* L. *Agronomy* 8:186. 10.3390/agronomy8090186

[B22] Li H. Huang W. Liu Z. W. Wang Y. X. Zhuang J. (2016). Transcriptome-based analysis of Dof family transcription factors and their responses to abiotic stress in tea plant (*Camellia sinensis*). *Int. J. Genom.* 2016:5614142. 10.1155/2016/5614142 27872842 PMC5107859

[B23] Li H. Wang Y. Wu M. Li L. Li C. Han Z. (2017). Genome-wide identification of AP2/ERF transcription factors in cauliflower and expression profiling of the ERF family under salt and drought stresses. *Front. Plant Sci.* 8:946. 10.3389/fpls.2017.00946 28642765 PMC5462956

[B24] Li W. H. (1993). Unbiased estimation of the rates of synonymous and nonsynonymous substitution. *J. Mol. Evol.* 36 96–99. 8433381 10.1007/BF02407308

[B25] Li W. H. Yang J. Gu X. (2005). Expression divergence between duplicate genes. *Trends Genet.* 21 602–607. 10.1016/j.tig.2005.08.006 16140417

[B26] Li Y. Xiao J. Wu J. Duan J. Liu Y. Ye X. (2012). A tandem segmental duplication (TSD) in green revolution gene *Rht-D1b* region underlies plant height variation. *New Phytol.* 196 282–291. 10.1111/j.1469-8137.2012.04243.x 22849513

[B27] Lijavetzky D. Carbonero P. Vicente-Carbajosa J. (2003). Genome-wide comparative phylogenetic analysis of the rice and *Arabidopsis* Dof gene families. *BMC Evol. Biol.* 3:17. 10.1186/1471-2148-3-17 12877745 PMC184357

[B28] Liu J. J. Ekramoddoullah A. K. (2009). Identification and characterization of the WRKY transcription factor family in *Pinus monticola*. *Genome* 52 77–88. 10.1139/G08-106 19132074

[B29] Liu X. Liu Z. Hao Z. Chen G. Qi K. Zhang H. (2020). Characterization of Dof family in *Pyrus bretschneideri* and role of *PbDof9.2* in flowering time regulation. *Genomics* 112 712–720. 10.1016/j.ygeno.2019.05.005 31078718

[B30] Livak K. J. Schmittgen T. D. (2001). Analysis of relative gene expression data using real-time quantitative PCR and the 2^–Δ^ ^Δ^ ^CT^ method. *Methods* 25 402–408. 10.1006/meth.2001.1262 11846609

[B31] Ma J. Li M. Y. Wang F. Tang J. Xiong A. S. (2015). Genome-wide analysis of Dof family transcription factors and their responses to abiotic stresses in Chinese cabbage. *BMC Genom.* 16:33. 10.1186/s12864-015-1242-9 25636232 PMC4320540

[B32] Makova K. D. Li W. H. (2003). Divergence in the spatial pattern of gene expression between human duplicate genes. *Genome Res.* 13 1638–1645. 10.1101/gr.1133803 12840042 PMC403737

[B33] Molina-Hidalgo F. J. Medina-Puche L. Canete-Gomez C. Franco-Zorrilla J. M. Lopez-Vidriero I. Solano R. (2017). The fruit-specific transcription factor *FaDOF2* regulates the production of eugenol in ripe fruit receptacles. *J. Exp. Bot.* 68 4529–4543. 10.1093/jxb/erx257 28981772

[B34] Moore R. C. Purugganan M. D. (2005). The evolutionary dynamics of plant duplicate genes. *Curr. Opin. Plant Biol.* 8 122–128. 10.1016/j.pbi.2004.12.001 15752990

[B35] Nan H. Gao L. Z. (2019). Genome-wide analysis of *WRKY* genes and their response to hormone and mechanic stresses in carrot. *Front. Genet.* 10:363. 10.3389/fgene.2019.00363 31191596 PMC6504813

[B36] Raymond O. Gouzy J. Just J. Badouin H. Verdenaud M. Lemainque A. (2018). The *Rosa* genome provides new insights into the domestication of modern roses. *Nat. Genet.* 50 772–777. 10.1038/s41588-018-0110-3 29713014 PMC5984618

[B37] Renau-Morata B. Molina R. V. Carrillo L. Cebolla-Cornejo J. Sánchez-Perales M. Pollmann S. (2017). Ectopic expression of *CDF3* genes in tomato enhances biomass production and yield under salinity stress conditions. *Front. Plant Sci.* 8:660. 10.3389/fpls.2017.00660 28515731 PMC5414387

[B38] Sasaki N. Matsumaru M. Odaira S. Nakata A. Nakata K. Nakayama I. (2015). Transient expression of tobacco BBF1-related Dof proteins, BBF2 and BBF3, upregulates genes involved in virus resistance and pathogen defense. *Physiol. Mol. Plant Pathol.* 89 70–77. 10.1016/j.pmpp.2014.12.005

[B39] Tian X. Wang Z. Zhang Q. Ci H. Wang P. Yu L. (2018). Genome-wide transcriptome analysis of the salt stress tolerance mechanism in *Rosa chinensis*. *PLoS One* 13:e0200938. 10.1371/journal.pone.0200938 30048505 PMC6062038

[B40] Tian X. Wang Z. Zhang Q. Ci H. Wang P. Yu L. (2019). Identification of salt stress response genes in *Rosa chinensis* leaves by comparative RNA-seq analysis of transcriptome dynamics. *Russ. J. Plant Physl.* 66 119–127. 10.1134/s1021443719010175

[B41] Wang M. Vannozzi A. Wang G. Liang Y. H. Tornielli G. B. Zenoni S. (2014). Genome and transcriptome analysis of the grapevine (*Vitis vinifera* L.) *WRKY* gene family. *Hortic. Res.* 1:14016. 10.1038/hortres.2014.16 26504535 PMC4596322

[B42] Wang S. Bai Y. Li P. Yang L. Wang X. (2019). Genome-wide identification and expression analysis of the dof (DNA binding with one finger) protein family in monocot and dicot species. *Physiol. Mol. Plant Pathol.* 108:101431. 10.1016/j.pmpp.2019.101431

[B43] Wray G. A. Hahn M. W. Abouheif E. Balhoff J. P. Pizer M. Rockman M. V. (2003). The evolution of transcriptional regulation in eukaryotes. *Mol. Biol. Evol.* 20 1377–1419. 10.1093/molbev/msg140 12777501

[B44] Wu Z. Cheng J. Cui J. Xu X. Liang G. Luo X. (2016). Genome-wide identification and expression profile of Dof transcription factor gene family in pepper (*Capsicum annuum* L.). *Front. Plant Sci.* 7:574. 10.3389/fpls.2016.00574 27200047 PMC4850169

[B45] Xiao Y. Zhou L. Lei X. Cao H. Wang Y. Dou Y. (2017). Genome-wide identification of *WRKY* genes and their expression profiles under different abiotic stresses in *Elaeis guineensis*. *PLoS One* 12:e0189224. 10.1371/journal.pone.0189224 29228032 PMC5724828

[B46] Yanagisawa S. (2001). The transcriptional activation domain of the plant-specific Dof1 factor functions in plant, animal, and yeast cells. *Plant Cell Physiol.* 42 813–822. 10.1093/pcp/pce105 11522906

[B47] Yanagisawa S. (2002). The Dof family of plant transcription factors. *Trends Plant Sci.* 7 555–560. 10.1016/S1360-1385(02)02362-2 12475498

[B48] Yanagisawa S. Izui K. (1993). Molecular cloning of two DNA-binding proteins of maize that are structurally different but interact with the same sequence motif. *J. Biol. Chem.* 268 16028–16036. 10.1016/S1360-1385(02)02362-2 8340424

[B49] Yanagisawa S. Schmidt R. J. (1999). Diversity and similarity among recognition sequences of Dof transcription factors. *Plant J.* 17 209–214. 10.1046/j.1365-313X.1999.00363.x 10074718

[B50] Yang Q. Chen Q. Zhu Y. Li T. (2018). Identification of *MdDof* genes in apple and analysis of their response to biotic or abiotic stress. *Funct. Plant Biol.* 45 528–541. 10.1071/FP17288 32290992

[B51] Yang Z. (2007). PAML 4: phylogenetic analysis by maximum likelihood. *Mol. Biol. Evol.* 24 1586–1591. 10.1093/molbev/msm088 17483113

[B52] Yang Z. H. Bielawski J. P. (2000). Statistical methods for detecting molecular adaptation. *Trends Ecol. Evol.* 15 496–503. 11114436 10.1016/S0169-5347(00)01994-7 PMC7134603

[B53] Yokoya K. (2000). Nuclear DNA amounts in Roses. *Ann. Bot.* 85 557–561. 10.1006/anbo.1999.1102

[B54] Zhang J. (2003). Evolution by gene duplication: an update. *Trends Ecol. Evol.* 18 292–298. 10.1016/s0169-5347(03)00033-8

[B55] Zhang Z. Yuan L. Liu X. Chen X. Wang X. (2018). Evolution analysis of Dof transcription factor family and their expression in response to multiple abiotic stresses in *Malus domestica*. *Gene* 639 137–148. 10.1016/j.gene.2017.09.039 28986315

[B56] Zou Z. Zhang X. (2019). Genome-wide identification and comparative evolutionary analysis of the Dof transcription factor family in physic nut and castor bean. *PeerJ* 7:e6354. 10.7717/peerj.6354 30740272 PMC6368027

[B57] Zou Z. Zhu J. Zhang X. (2019). Genome-wide identification and characterization of the Dof gene family in cassava (*Manihot esculenta*). *Gene* 687 298–307. 10.1016/j.gene.2018.11.053 30472376

